# Supplementary material for: Quantifying the global climate feedback from energy-based adaptation
Source: Nat Commun. 2025 Apr 26;16:3928. doi: 10.1038/s41467-025-59201-7 (PMC12032092; doi:10.1038/s41467-025-59201-7)
Supplement: Supplementary file 1 — Supplementary Information [file 41467_2025_59201_MOESM1_ESM.pdf]

Supplementary Information to "Quantifying the global climate feedback from energy-based adaptation"

Alexander C. Abajian,[1*] Tamma Carleton,[2,3*] Kyle C. Meng,[1,3,4*] Olivier Deschênes[1,3]

[1]Department of Economics, University of California, Santa Barbara, CA 93106 USA

[2]Department of Agricultural and Resource Economics, University of California, Berkeley, CA 94720 USA

[3]National Bureau of Economic Research, Cambridge, MA 02138 USA

[4]Bren School of Environmental Science and Management, University of California, Santa Barbara, CA 94720 USA

[*]To whom correspondence should be addressed.

E-mails: alexander_abajian@ucsb.edu, tcarleton@berkeley.edu, kmeng@bren.ucsb.edu.

Contents

Supplementary Note 1: CAF decomposition by fuel **3**

Supplementary Note 2: Accounting for the extensive margin of adaptation **5**

Supplementary Note 3: Solving for global emissions factors **7**

Supplementary Note 4: Case study of India **9**

Supplementary Note 5: Robustness of the Climate Adaptation Feedback to the omission of individual countries **11**

Supplementary References **13**

Supplementary Note 1: CAF decomposition by fuel

Supplementary Figs. 1 and 2 decompose adaptation-induced cumulative country-level emissions in 2099 (shown in main text Figure 4a) into two types of fuels: electricity (Fig. 1) and all other fuels (Fig. 2). Both figures display fuel-specific cumulative emissions due to energy-based adaptation to climate change at the country level under our baseline SSP2-RCP8.5 scenario.

Supplementary Fig. 1 shows that in most countries, climate change is projected to increase demand for electricity relative to a counterfactual where local temperature distributions were held at the 2001-10 average level, raising electricity-driven CO_2 emissions. Countries in dark red generate the most emissions due to electricity-based adaptations to climate change. However, Supplementary Fig. 2 shows that all countries are forecast to experience declines in emissions from declining demand for other fuels under climate change. This latter force offsets the former in most countries, leading to net negative effects of adaptation on total CO_2 emissions for most of the world (shown in main text Figure 4a), and making our baseline CAF estimate negative.

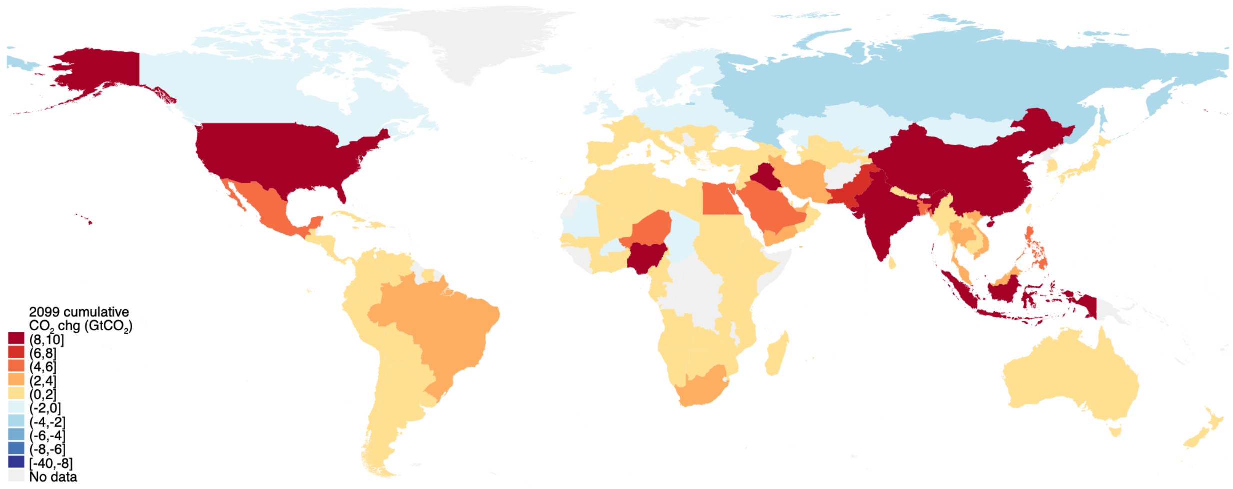

Supplementary Figure 1: Cumulative emissions in 2099 induced by electricity-based adaptation. The map displays the country-level cumulative adaptation-induced carbon dioxide (CO_2) emissions in 2099 due to the electricity component of the Climate Adaptation Feedback (CAF). This is measured in gigatonnes of carbon dioxide ($GtCO_2$), as in main text Figure 4a, and corresponds to the electricity component of equation (3) in the Methods section of the main text when evaluated for 2099.

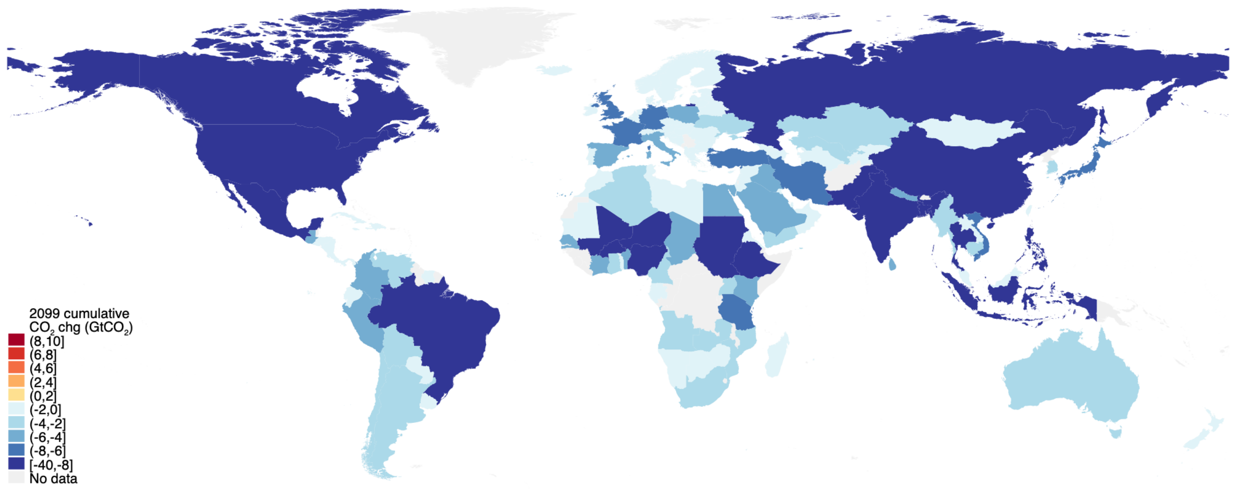

Supplementary Figure 2: Cumulative emissions in 2099 induced by other fuels-based adaptation. The map displays the country-level cumulative adaptation-induced carbon dioxide (CO_2) emissions in 2099 due to the other fuels component of the Climate Adaptation Feedback (CAF). This is measured in gigatonnes of carbon dioxide ($GtCO_2$), as in main text Figure 4a, and corresponds to the electricity component of equation (3) in the Methods section of the main text when evaluated for 2099.

Supplementary Note 2: Accounting for the extensive margin of adaptation

This section demonstrates the importance of the extensive margin of adaptive energy use for constructing an accurate estimate of the Climate Adaptation Feedback (CAF). In the baseline CAF calculation shown in the main text, estimates of energy demand changes under climate change are constructed using dose-response functions from ref. (1) that account for both long-term adjustments due to gradually evolving climate (e.g., the increased adoption of air conditioning in today's temperate climates as they warm in the future) and due to growing incomes (e.g., the increased adoption of air conditioning in today's developing economies as they experience economic growth in the future). To account for such extensive margin adjustments, ref. (1) estimate regression models that interact long-run average climate and long-run average GDP per capita with local short-run variation in the weather (we direct the reader to the Methods section in ref. (1) as well as their Supplementary Information for details), an approach analogous to the two-step method used in ref. (2). These econometric estimates are then used in combination with climate and socioeconomic projections to estimate how energy demand responses to daily weather realizations will change due to extensive margin adjustments.

Here, we quantify the importance of accounting for such extensive margin effects by computing the CAF using a set of demand responses from ref. (1) that do not include extensive margin adjustments. Specifically, Supplementary Fig. 3 displays the baseline CAF for the SSP2-RCP4.5 scenario in a solid grey line, as well as a counterfactual CAF in a dashed grey line for the same scenario but without extensive margin adjustments. We find that by 2099, the CAF would be only 20 percent as large without these dynamic extensive margin adjustments.

The treatment of the extensive margin for adaptation in ref. (1) is standard in the literature (e.g., refs. (2; 3)). However, this approach is not without its limitations. In particular, future technological innovation and/or rates of adoption may evolve in ways that are not driven by the two factors (long-run climate and income) used in the estimations of refs. (1), (2), and (3), among others. For example, if the relative price of cooling technologies falls in the future, our CAF estimate may be too low and understate the increase in future cooling demand and emissions driven by warming climates. However, it is also possible that energy efficiency in cooling and/or heating technologies may improve with economic growth at rates faster than those observed historically, pushing the CAF in the opposite direction.

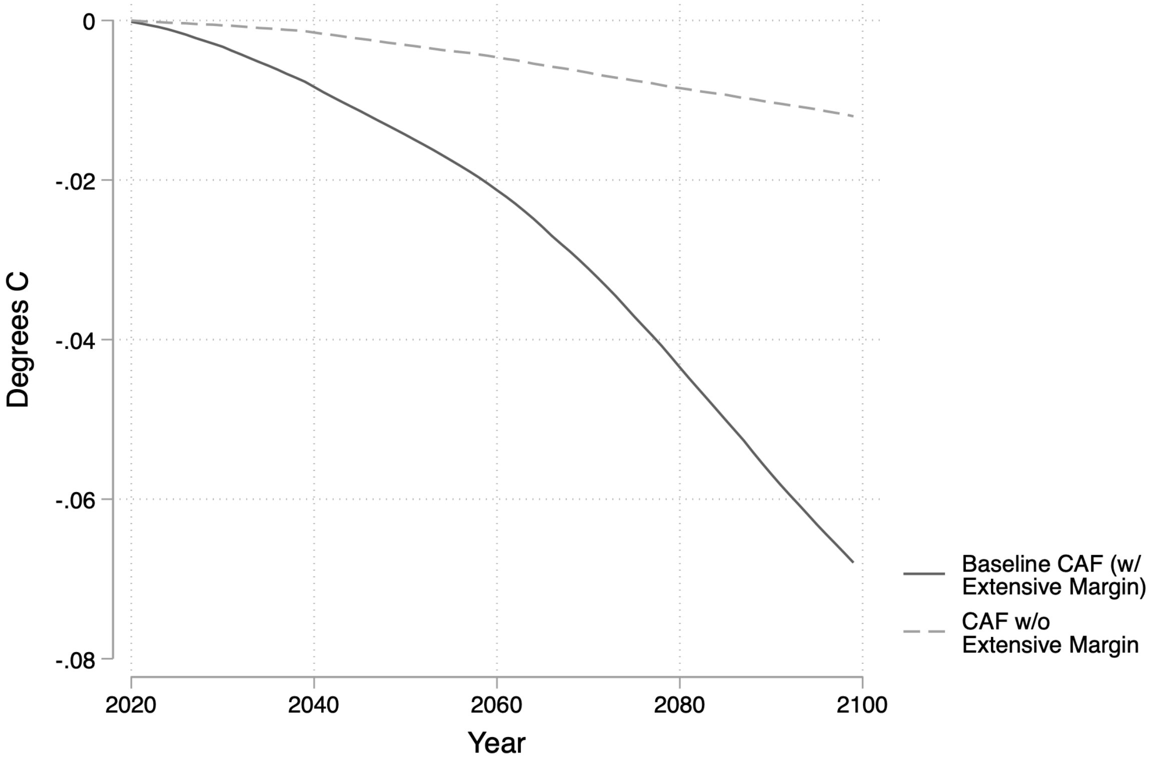

Supplementary Fig. 3: Computing the Climate Adaptation Feedback (CAF) with versus without an extensive margin of adaptation. This figure compares the baseline CAF from the main text (solid line) to an alternative in which all energy demand responses to future temperature realizations are fixed at 2001-2010 levels (dashed line). This alternative shows what the CAF would be were extensive margin adjustments in energy demand responses to temperature ignored. Both estimates use the scenario generated by pairing Shared Socioeconomic Pathway 2 with Representative Concentration Pathway 8.5.

Supplementary Note 3: Solving for global emissions factors

The long dashed lime green line in Figure 3a of the main text displays an alternative estimate of the CAF for SSP2-RCP8.5 in which future fuel-specific emissions factors are adjusted to decay in the future based on observed historical rates. As emissions from both fuels have declined on a unit basis throughout the 21st century, for this sensitivity analysis we estimate the CAF assuming historical trends continue through 2099. To calculate an appropriate degree of decay for each fuel, we estimate the annual rate of change in global average emissions factors (in units of $tCO_2 \times kWh^{-1}$ of final energy use) between 2000 and 2018 using data on emissions and final energy consumption from the IEA (4; 5).

Supplementary Fig. 4 displays a time series of annual global average emissions factors for electricity (solid line) and other fuels (dashed line) for the 2000-2018 period calculated using data from the International Energy Agency (4). To calculate average rates of change over time, for each fuel h, we estimate the following exponential decay coefficient using equation (1):

$$\Delta \ln F_t^h = \gamma_h + \varepsilon_{h,t} \tag{1}$$

where $\Delta \ln F_t^h$ is the change in the (log) global emissions factor for fuel h from the previous year and $\varepsilon_{h,t}$ are random error terms. The estimated global decay factors $\hat{\gamma}_h$ are $-.0008$ and $-.009$ for other fuels and electricity, respectively. We use these estimates to generate a future pathway for each country-by-fuel specific factor using equation (2):

$$F_{i,t}^h = \exp\Big\{\hat{\gamma}_h(t-2020)\Big\}\bar{F}_i^h \tag{2}$$

where $\bar{F}_i^h$ denotes the original 2010-18 emissions factors calculated based on country-level data between 2010-18 and used for the baseline CAF.

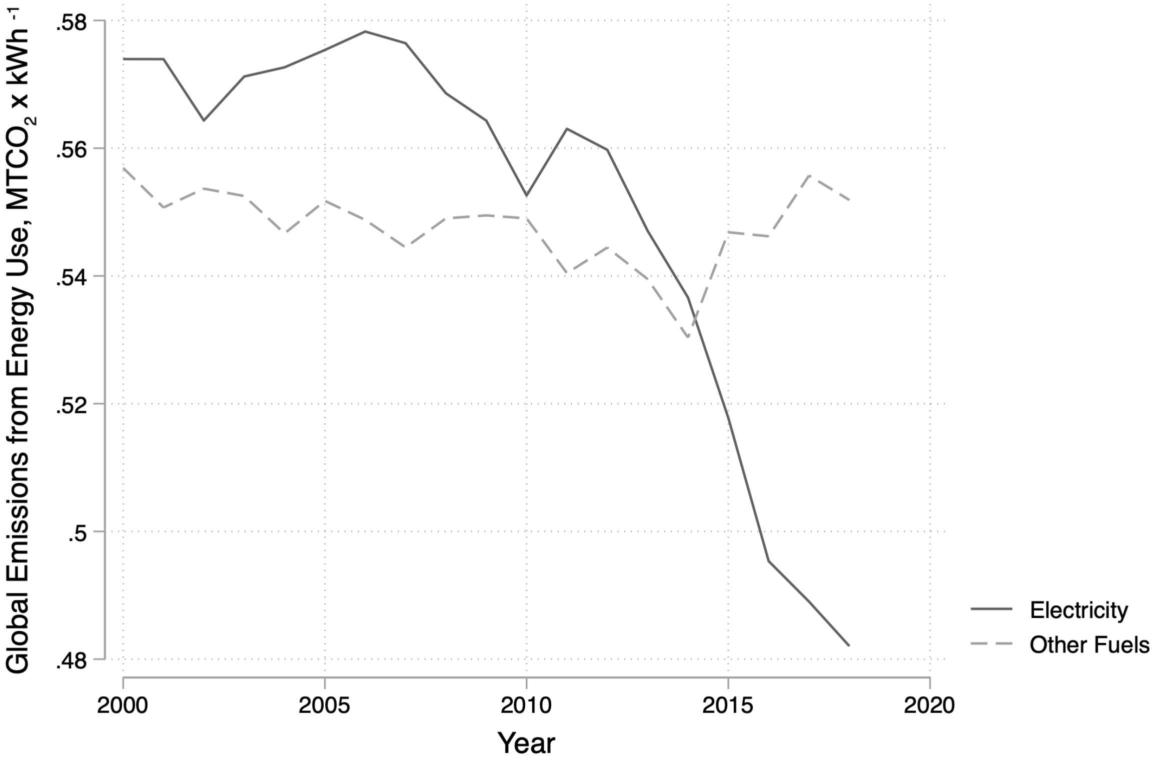

Supplementary Fig. 4: Recent changes in global emissions factors. This figure shows annual global emissions factors for electricity (solid line) and other fuels (dashed line) between 2000 and 2018 calculated using data from the International Energy Agency.

Supplementary Note 4: Case study of India

Figure 4a in the main text shows that India exhibits large adaptation-induced declines in CO_2 emissions by end-of-century, despite facing substantial increases in exposure to extreme heat in future years (6). In this section, we decompose this result, showing that it is driven both by heterogeneous demand responses across electricity (largely used for cooling) and other fuels (largely used for heating), as well as heterogeneous emissions intensities of these two fuel types. Specifically, we show our forecasts for how adaptation to climate change will affect energy demand (Supplementary Fig. 5) and in turn, emissions (Supplementary Fig. 6) for each fuel type for India under our baseline SSP2-RCP8.5 scenario.

Supplementary Fig. 5 shows that by 2099, adaptation to climate change will induce an additional 5 Exajoules (EJs; billions of Gigajoules) of electricity demand relative to a counterfactual demand without climate change induced adaptation. This effect is almost 50% larger in magnitude than total annual electricity consumption in India today (c.f. Figure 2b in ref. (1)), representing a large increase in demand for cooling. However, Supplementary Fig. 5 also shows that the increased demand for electricity is offset by declining demand for other fuels in a future under climate change. Specifically, other fuels consumption is projected to fall by about 7 EJs in India by 2099. This reduction is sizable, but also plausible, when compared to current Indian fossil fuel use outside the electricity sector. Of the approximately 40 EJ of primary energy use in India today, at least 7.5 EJ is biomass, an energy source used almost exclusively for indoor heating and cooking, activities that are sensitive to colder temperatures (7; 8). The forecast annual reductions in other fuels consumption in 2099 due to adaptation are thus comparable to a decline in current biomass consumption levels by over 90 percent. Of course, other fuel sources – such as coal, oil, and natural gas – are also used for indoor heating and cooking in India, and will additionally contribute to declining other fuels consumption under warmer future climates.

This result that other fuels demand declines by more than electricity demand increases is driven by a key finding of the ref. (1) paper, which is that for lower-income regions, electricity demand is essentially unresponsive to high temperatures, while other fuels (including biomass burning, among other sources) *are* used in these regions to respond to moderate to cold conditions. Even though India is projected to experience substantial economic growth under the SSP2 socioeconomic scenario, its relatively low level of baseline income inhibits a stronger electricity demand response (1). This larger (in magnitude of EJs) demand effect on other fuels is compounded by the fact that on a unit basis, other fuels consumption in India is more carbon intensive than is electricity. Supplementary Fig. 6 shows the associated effects of changes in demand on changes in emissions. India's other fuels consumption emits 1.38

$kgCO_2 \times kWh^{-1}$ of energy produced, almost double the factor of 0.8 $kgCO_2 \times kWh^{-1}$ for electricity. These two facts combine to produce the large (negative) change in cumulative emissions for India in 2099, shown in main text Figure 4a.

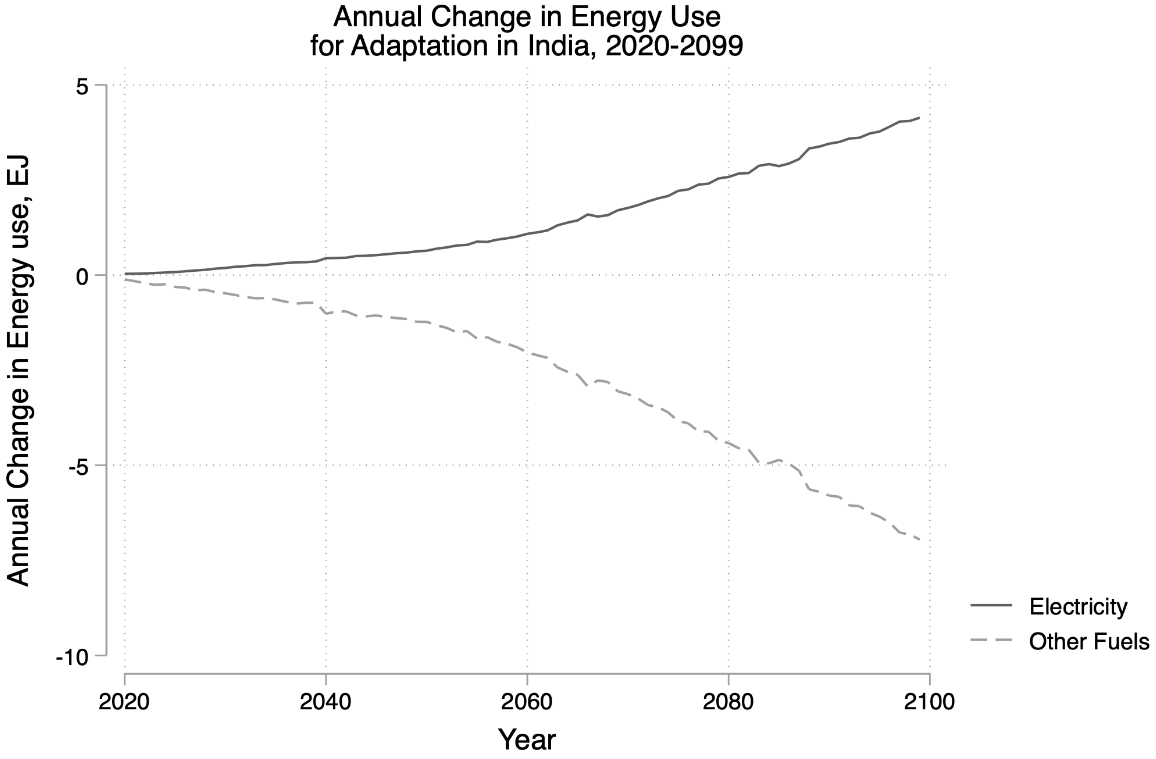

Supplementary Fig. 5: Change in energy use due to adaptation to climate change in India. This figure shows the mean value of projected annual changes in energy demand from electricity (solid line) and other fuels (dashed line) due to adaptation to climate change in India between 2020 and 2099. Values are averaged over projections under the scenario generated by pairing Shared Socioeconomic Pathway 2 with Representative Concentration Pathway 8.5.

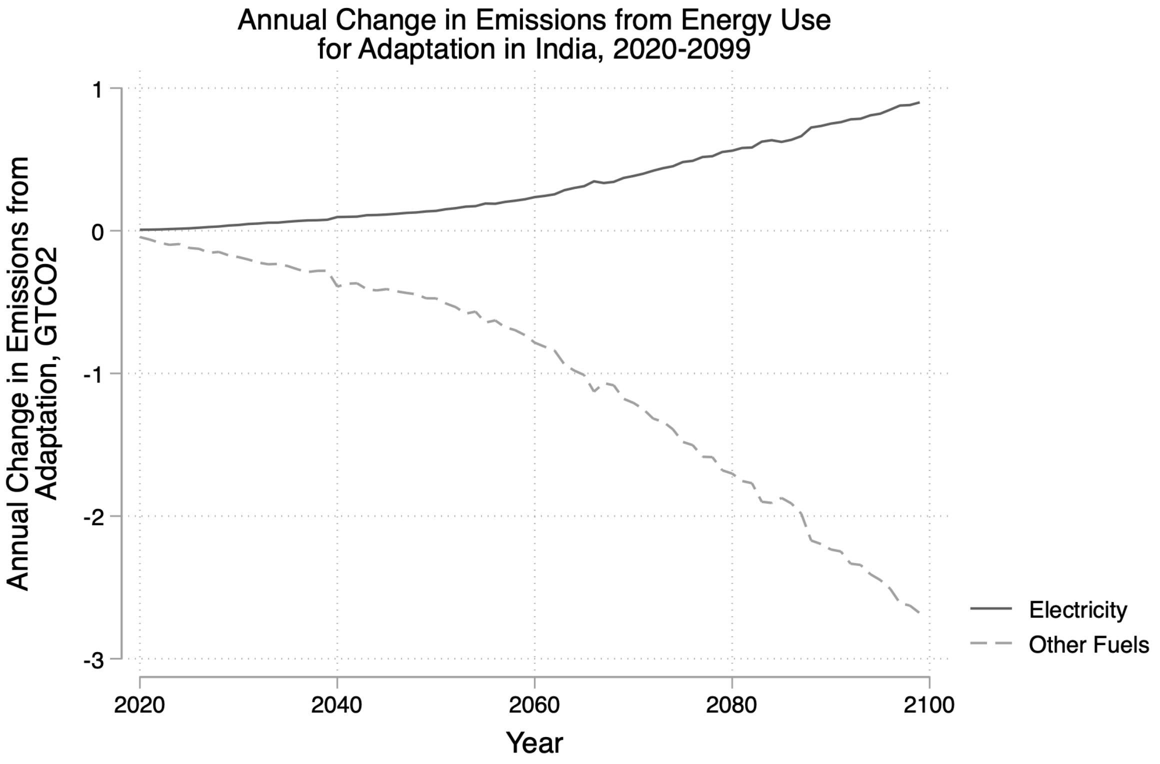

Supplementary Fig. 6: Change in emissions due to energy-based adaptation to climate change in India. This figure converts the energy use changes in Supplementary Fig. 5 into carbon dioxide emissions using emissions factors for India. Carbon dioxide (CO_2) emissions due to energy-based adaptation are shown for electricity (solid line) and other fuels (dashed line).

Supplementary Note 5: Robustness of the Climate Adaptation Feedback to the omission of individual countries

Here we conduct an analysis to evaluate whether individual locations are driving our final CAF result. To do so, we recalculate the cumulative global change in emissions due to adaptive energy use repeatedly, each time removing the emissions effects of adaptive energy use from one country before re-computing global emissions changes. Supplementary Fig. 7 shows the result, plotting the distribution of changes in cumulative emissions in 2099 under our baseline SSP2-RCP8.5 scenario over simulations in which each of the $N = 142$ countries in our sample are removed. The dashed line is our baseline estimate (an emissions reduction of 195 $GTCO_2$) which includes all 142 countries featured in our main analysis. This figure shows that all leave-one-out estimates of cumulative emissions in 2099 are well below zero and differ from the full-country estimate by at most 20%.

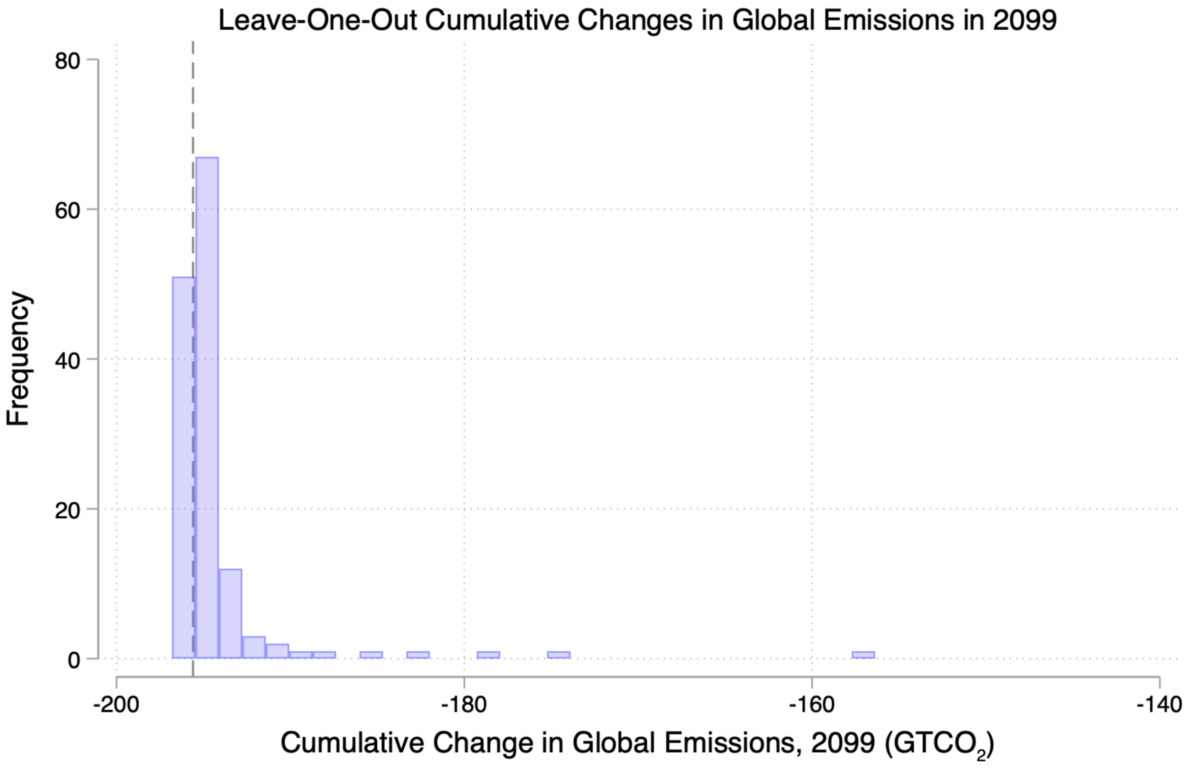

Supplementary Fig. 7: Distribution of emissions changes due to adaptive energy use in a leave-one-country-out sensitivity analysis. Distribution of global emissions changes due to adaptive energy use in 2099 over 142 simulations, each of which removes the adaptive emissions from one country. The full-country estimate from the main text is indicated by the vertical dashed line. Results shown are from our baseline scenario generated by pairing Shared Socioeconomic Pathway 2 with Representative Concentration Pathway 8.5.

Supplementary References

[1] Rode, A. *et al.* Estimating a social cost of carbon for global energy consumption. *Nature* **598**, 308–314 (2021).

[2] Auffhammer, M. Climate adaptive response estimation: Short and long run impacts of climate change on residential electricity and natural gas consumption. *Journal of Environmental Economics and Management* **114**, 102669 (2022).

[3] De Cian, E. & Sue Wing, I. Global energy consumption in a warming climate. *Environmental and resource economics* **72**, 365–410 (2019).

[4] IEA. Emissions factors. Tech. Rep., International Energy Agency (IEA) (2021). URL `https://www.iea.org/data-and-statistics/data-product/emissions-factors-2021`. All Rights Reserved.

[5] IEA. World energy balances 2021. Tech. Rep., International Energy Agency (IEA) (2022). URL `https://www.iea.org/data-and-statistics/data-product/world-energy-balances`. All Rights Reserved.

[6] Krishnan, R. *et al.* Introduction to climate change over the indian region. *Assessment of climate change over the indian region: a report of the ministry of earth sciences (MoES), Government of India* 1–20 (2020).

[7] IEA. India energy outlook 2021. Tech. Rep., International Energy Agency (IEA) (2021). URL `https://www.iea.org/reports/india-energy-outlook-2021`. All Rights Reserved.

[8] EIA. International energy database. Tech. Rep., United States Energy Information Administration (EIA) (2024). URL `https://www.eia.gov/international/data/world`.
