# Supplementary material for: Quantifying the global climate feedback from energy-based adaptation
Source: Nat Commun. 2025 Apr 26;16:3928. doi: 10.1038/s41467-025-59201-7 (PMC12032092; doi:10.1038/s41467-025-59201-7)
Supplement: Supplementary file 2 — Reporting Summary [file 41467_2025_59201_MOESM2_ESM.pdf]

nature portfolio

Corresponding author(s): Alexander Abajian, Tamma Carleton, Kyle Meng

Last updated by author(s): Feb 19, 2025

Reporting Summary

Nature Portfolio wishes to improve the reproducibility of the work that we publish. This form provides structure for consistency and transparency in reporting. For further information on Nature Portfolio policies, see our Editorial Policies and the Editorial Policy Checklist.

Statistics

For all statistical analyses, confirm that the following items are present in the figure legend, table legend, main text, or Methods section.

n/a	Confirmed	
☐	☒	The exact sample size (*n*) for each experimental group/condition, given as a discrete number and unit of measurement
☒	☐	A statement on whether measurements were taken from distinct samples or whether the same sample was measured repeatedly
☐	☒	The statistical test(s) used AND whether they are one- or two-sided<br>*Only common tests should be described solely by name; describe more complex techniques in the Methods section.*
☐	☒	A description of all covariates tested
☒	☐	A description of any assumptions or corrections, such as tests of normality and adjustment for multiple comparisons
☐	☒	A full description of the statistical parameters including central tendency (e.g. means) or other basic estimates (e.g. regression coefficient) AND variation (e.g. standard deviation) or associated estimates of uncertainty (e.g. confidence intervals)
☐	☒	For null hypothesis testing, the test statistic (e.g. *F*, *t*, *r*) with confidence intervals, effect sizes, degrees of freedom and *P* value noted<br>*Give P values as exact values whenever suitable.*
☒	☐	For Bayesian analysis, information on the choice of priors and Markov chain Monte Carlo settings
☒	☐	For hierarchical and complex designs, identification of the appropriate level for tests and full reporting of outcomes
☒	☐	Estimates of effect sizes (e.g. Cohen's *d*, Pearson's *r*), indicating how they were calculated

Our web collection on statistics for biologists contains articles on many of the points above.

Software and code

Policy information about availability of computer code

Data collection	Stata SE v. 18.5; Microsoft Excel Version 16.94 (25020927)
Data analysis	Stata SE v. 18.5; R 4.1.1; Microsoft Excel Version 16.94 (25020927)

For manuscripts utilizing custom algorithms or software that are central to the research but not yet described in published literature, software must be made available to editors and reviewers. We strongly encourage code deposition in a community repository (e.g. GitHub). See the Nature Portfolio guidelines for submitting code & software for further information.

Data

Policy information about availability of data

All manuscripts must include a data availability statement. This statement should provide the following information, where applicable:

- Accession codes, unique identifiers, or web links for publicly available datasets
- A description of any restrictions on data availability
- For clinical datasets or third party data, please ensure that the statement adheres to our policy

Emissions intensities and annual consumption at the country-by-fuel level are taken from the International Energy Agency's Emissions Intensities Report and World Energy Balance datasets, respectively. The raw IEA data are a subscription-only product that cannot be publicly shared. All other raw and processed data used in the main manuscript and supplementary information required for a bottom-up replication of all analysis are publicly available in the Zenodo repository associated with the paper at https://doi.org/10.5281/zenodo.10476310}.

Research involving human participants, their data, or biological material

Policy information about studies with human participants or human data. See also policy information about sex, gender (identity/presentation), and sexual orientation and race, ethnicity and racism.

Reporting on sex and gender	n/a
Reporting on race, ethnicity, or other socially relevant groupings	n/a
Population characteristics	n/a
Recruitment	n/a
Ethics oversight	n/a

Note that full information on the approval of the study protocol must also be provided in the manuscript.

Field-specific reporting

Please select the one below that is the best fit for your research. If you are not sure, read the appropriate sections before making your selection.

☐ Life sciences ☐ Behavioural & social sciences ☒ Ecological, evolutionary & environmental sciences

For a reference copy of the document with all sections, see nature.com/documents/nr-reporting-summary-flat.pdf

Ecological, evolutionary & environmental sciences study design

All studies must disclose on these points even when the disclosure is negative.

Study description	This study forecasts the carbon emissions resulting from projected changes in global non-transport energy use.
Research sample	Forecasts from Rode et al. (2021) along with data from the International Energy Agency (IEA).
Sampling strategy	The 5th through the 95th percentile findings from Rode et al. (2021) along with the universe of emissions data from IEA.
Data collection	Demand forecasts are constructed by Rode et al. (2021) and emissions factors are collected by the IEA.
Timing and spatial scale	A country-level resolution at the global scale from 2020 through 2099.
Data exclusions	Countries for which we cannot construct emissions factors or do not possess demand forecasts are excluded.
Reproducibility	A number of data inputs are proprietary and must be purchased from the IEA and used according to the associated EULA. All other raw and processed data used in the main manuscript and supplementary information required for a bottom-up replication of all analysis are publicly available in the Zenodo repository associated with the paper at https://doi.org/10.5281/zenodo.10476310. The computer code required to run these analysis may be found at https://doi.org/10.5281/zenodo.14894953.
Randomization	n/a
Blinding	n/a

Did the study involve field work? ☐ Yes ☒ No

Reporting for specific materials, systems and methods

We require information from authors about some types of materials, experimental systems and methods used in many studies. Here, indicate whether each material, system or method listed is relevant to your study. If you are not sure if a list item applies to your research, read the appropriate section before selecting a response.

Materials & experimental systems

n/a	Involved in the study
☒	☐ Antibodies
☒	☐ Eukaryotic cell lines
☒	☐ Palaeontology and archaeology
☒	☐ Animals and other organisms
☒	☐ Clinical data
☒	☐ Dual use research of concern
☒	☐ Plants

Methods

n/a	Involved in the study
☒	☐ ChIP-seq
☒	☐ Flow cytometry
☒	☐ MRI-based neuroimaging

Plants

Seed stocks	n/a
Novel plant genotypes	n/a
Authentication	n/a
